# High prevalence of vaccine‐preventable anal human papillomavirus infections is associated with HIV infection among gay, bisexual, and men who have sex with men in Nairobi, Kenya

**DOI:** 10.1002/cam4.6008

**Published:** 2023-05-04

**Authors:** Myo Minn Oo, Samantha Moore, Suzanne Gibbons, Wendy Adhiambo, Peter Muthoga, Naomi Siele, Maureen Akolo, Henok Gebrebrhan, Aida Sivro, Blake T. Ball, Robert R. Lorway, Alberto Severini, Joshua Kimani, Lyle R. McKinnon

**Affiliations:** ^1^ Department of Medical Microbiology and Infectious Diseases University of Manitoba Winnipeg Manitoba Canada; ^2^ Institute for Global Public Health (IGPH) University of Manitoba Winnipeg Manitoba Canada; ^3^ JC Wilt Infectious Disease Research Centre National Microbiology Laboratory, Public Health Agency of Canada Winnipeg Manitoba Canada; ^4^ University of Nairobi Institute of Tropical and Infectious Diseases, University of Nairobi Nairobi Kenya; ^5^ Department of Medical Microbiology University of KwaZulu‐Natal Durban South Africa; ^6^ Centre for the AIDS Programme of Research in South Africa (CAPRISA) Durban South Africa

**Keywords:** anal HPV, bisexual, gay and men who have sex with men (gbMSM), HIV/STI, HPV genotypes, HPV vaccination, Kenya

## Abstract

**Background:**

Human papillomavirus (HPV) infection is associated with anal cancers and is more prevalent in gay, bisexual, and men who have sex with men (gbMSM), partly due to their vulnerability to HIV infection. Baseline HPV genotype distributions and risk factors can inform the design of next‐generation HPV vaccines to prevent anal cancer.

**Methods:**

A cross‐sectional study was conducted among gbMSM receiving care at a HIV/STI clinic in Nairobi, Kenya. Anal swabs were genotyped using a Luminex microsphere array. Multiple logistic regression methods were used to identify risk factors for four HPV outcomes (any HPV, any HR‐HPV, and 4‐ and 9‐valent vaccine‐preventable HPVs).

**Results:**

Among 115 gbMSM, 51 (44.3%) were HIV‐infected. Overall HPV prevalence was 51.3%; 84.3% among gbMSM living with HIV and 24.6% among gbMSM without HIV (*p* < 0.001). One‐third (32.2%) had HR‐HPV and the most prevalent vaccine‐preventable HR‐HPV genotypes were 16, 35, 45, and 58. HPV‐18 was uncommon (*n* = 2). The 9‐valent Gardasil vaccine would have prevented 61.0% of HPV types observed in this population. In multivariate analyses, HIV status was the only significant risk factor for any HPV (adjusted odds ratio [aOR]:23.0, 95% confidence interval [95% CI]: 7.3–86.0, *p* < 0.001) and for HR‐HPV (aOR: 8.9, 95% CI: 2.8–36.0, *p* < 0.001). Similar findings were obtained for vaccine‐preventable HPVs. Being married to a woman significantly increased the odds of having HR‐HPV infections (aOR: 8.1, 95% CI: 1.6–52.0, *p* = 0.016).

**Conclusions:**

GbMSM living with HIV in Kenya are at higher risk of anal HPV infections including genotypes that are preventable with available vaccines. Our findings support the need for a targeted HPV vaccination campaign in this population.

## Graphical abstract

This study found that HIV‐infected gbMSMs had a significantly higher prevalence of HPV than those without HIV. The most common vaccine‐preventable HR‐HPV genotypes were 16, 35, 45, and 58. The 9‐valent Gardasil vaccine could have prevented 61.0% of HPV types observed in this population.

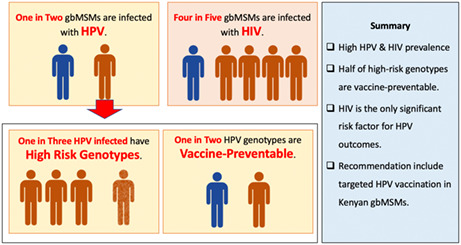

## 1 INTRODUCTION

Globally, human papillomavirus (HPV) causes anal cancer in an estimated number of 30,000 people and one‐third are in men.^1^ Anal cancer is rare in general population, but persistent anal infection with high‐risk HPVs can lead to increased risk of anal cancer.^2^, ^3^, ^4^ The burden of anal cancer in men has been increasing in the past decade.^5^ Gay, bisexual, and men who have sex with men (gbMSM) are more likely to have HPV infection than women and heterosexual men, partly due to their increased risk of anal HPV infection.^6^ Moreover, as HIV infection is a known risk factor for anal cancer in men,^7^, ^8^ gbMSM living with HIV are at an even greater risk of multiple HPV infections and developing cancerous lesions.^9^ A recent systematic review estimated an overall HPV prevalence difference of 11.1% between gbMSM living with HIV and without HIV.^10^ Anal cancer incidence rate is also five times higher in gbMSM living with HIV than in HIV‐negative counterparts.^11^

Among more than 50 types of HPV reported to be spread through sexual contact and that infect primarily the anogenital areas, 13 high‐risk HPV (HR‐HPV; HPV16, 18, 31, 33, 35, 39, 45, 51, 52, 56, 58, 59, and 68) have been identified as carcinogenic,^3^ and HPV16 is more frequently identified in HPV‐related anal cancer than in cervical cancer.^2^, ^12^ However, HPV prevalence and genotype distributions appear to vary both geographically and temporally. Studies from several countries in sub‐Saharan Africa including South Africa, the Central African Republic, and Nigeria indicated a higher prevalence of anal HR‐HPV infection, ranging from 57.6% to 82.7% among gbMSM, especially in HIV‐positive groups.^13^, ^14^, ^15^ Profiles of anal HR‐HPV genotypes in Africa were nonetheless diverse and the study from Nigeria also reported an unusual and unique profile of HR‐HPV 35 being the predominant one.^13^ These data suggest the importance of characterizing HPV epidemiology and genotype distribution across different regions of Africa to further inform local HPV prevention strategies.

HPV vaccination and the prevention and control of HIV infection are two primary prevention strategies for mitigating anal cancer. The HPV vaccine is considered to be the most efficient biological intervention to prevent HPV infection and subsequent diseases.^16^, ^17^ While HPV vaccination has been widely incorporated into routine national schedules for adolescent girls and young women (AGYW) in at least 100 countries worldwide, most countries, including Kenya, do not include gbMSM in their HPV vaccination programs, albeit similar effectiveness against HPV infection and subsequent conditions in males.^17^ Moreover, current global immunization guidelines now recommend HPV vaccination for men up to 26 years of age, as well as for gbMSM living with HIV up to and including 45 years of age.^18^, ^19^ In view of the increasing HPV‐related anal cancer burden on gbMSM and its similarities to cervical cancer, some have proposed an urgent call for Kenya to include the gbMSM population in the national HPV immunization program.^20^ Hence, this study assessed HPV prevalence, characterized genotype distributions, and explored risk factors for HPV infections, with a particular focus on vaccine‐preventable HPV types.

## 2 MATERIALS AND METHODS

A cross‐sectional study was conducted in 2016 among gbMSM attending HIV/STI clinics in Nairobi, Kenya. These clinics provide HIV/STI care, in support of gbMSM in Nairobi County. Those who were males at birth, aged 18 years and above, and who reported a history of having sex with men were eligible for inclusion in the study. Following informed written consent, a trained counselor interviewed participants using a standardized questionnaire that captured sociodemographic characteristics, including age, education, marital status, as well as HIV‐related sexual risks information such as type and frequency of sexual behaviors with different types of partners, condom use, and anal douching. After the interviews, clinical data and biological samples including rectal swabs were collected by a trained clinician.

### 2.1 HPV testing and genotyping

Post‐enema rectal swabs from participants were tested at the JC Wilt Infectious Diseases Research Lab, National Microbiology Laboratory (NML; Winnipeg, Manitoba) using an in‐house microsphere genotyping assay that distinguishes 46 mucosal HPV types.^21^ HPV DNA was amplified by nested PCR using type‐common primers and typed by hybridization to HPV type‐specific probes coupled to sortable microspheres based on the Luminex technology, as previously described.^21^ According to the International Agency for Research on Cancer (IARC) nomenclature, there are 13 high‐risk types (HR‐HPV ‐16, ‐18, ‐31, ‐33, ‐35, ‐39, ‐45, ‐51, ‐52, ‐56, ‐58, ‐59, and ‐68), 6 genotypes reported as possibly carcinogenic (HPV‐26, ‐61, ‐66, ‐69, ‐73 and ‐82), 9 low‐risk (LR) types (LR‐HPV ‐6, ‐11, ‐40, ‐42, ‐43, ‐44, ‐53, ‐54 and ‐70), and other types of unknown risk.^3^, ^13^ HPV genotypes were also classified according to their coverage by the 4‐valent Gardasil vaccine, which is effective against HPV genotypes 6, 11, 16, and 18, or by the 9‐valent Gardasil vaccine, which is effective against HPV genotypes 6, 11, 16, 18, 31, 33, 45, 52, and 58.

### 2.2 HIV and STI testing

Rapid screening tests were used in the clinics to determine HIV‐1 infection status, as per Kenyan HIV testing guidelines. HIV‐positive results were confirmed via GS HIV Combo Ab/Ab EIA (Bio‐Rad, Hercules) and Avioq HIV‐q Microelisa System (Avioq Inc.) at the JC Wilt lab in Winnipeg, Manitoba. HIV viral loads were also quantified using the RealTime HIV‐1 viral load assay (Abbott) on an automated m2000 RealTime system (Promega). STIs including *Neisseria gonorrhea* were tested using the Seegene Anyplex 6‐plex kit from pre‐enema rectal swabs.

### 2.3 Variables and definitions

The primary outcomes of interest include prevalence of HPV infections, HR‐HPV, multiple HPV types including HPV‐16 and ‐18 coinfection, and any or multiple vaccine‐preventable HPV types. HR‐HPV infection was defined as having one or more HR‐HPV genotypes, regardless of being co‐infected with LR‐HPV genotypes. Sociodemographic and clinical variables included age, marital status (to a woman), level of education, HIV infection, ART status, CD4 count, CD4 percentage, viral load, and STI (none, gonorrhea, other). Additional sexual behaviors include engaging in sex work, duration of sex work, and number of sex partners (regular, casual, or transactional) in the past month with different types of sex (oral, insertive, receptive) with or without condom, and douching practice. Sexual partners were defined as either regular (having a stable sexual relationship with a male partner rather than one‐off or selling sex work), casual (single sexual encounter that does not encompass regular or transactional in nature), or transactional (selling sex in exchange of money, material support, or other benefits).

### 2.4 Statistical analyses

Data were cleaned and analyzed using R software version 4.2.1. Baseline characteristics were summarized by descriptive statistics, including frequency and proportion for categorical variables and median and interquartile range (IQR) for continuous variables. Based on sample distribution, age was categorized into two groups: ≤30 and 30+ years. The genotype‐specific prevalence was estimated and compared by HIV status and age group. The prevalence of having any HPV, any HR‐HPV, 9‐ and 4‐valent vaccine‐preventable HPV was compared by baseline characteristics. Their risk factors were also assessed using multiple logistic regression models and effect sizes were displayed as unadjusted (uORs) and adjusted odds ratios (aORs) as well as corresponding 95% confidence intervals (CI).

### 2.5 Ethical considerations

All participants gave informed consent prior to participation. This study was approved by the Kenyatta National Hospital Ethics Review Committee and the Institutional Review Board at the University of Manitoba.

## 3 RESULTS

### 3.1 Characteristics of the study population

**FIGURE 1:**
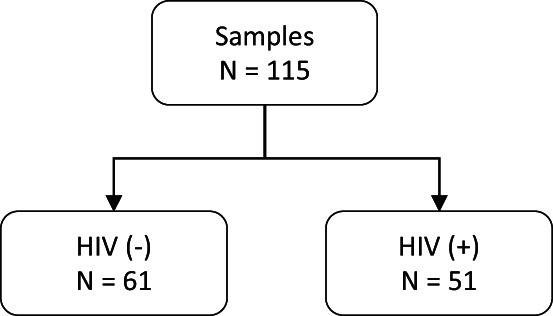
Sample flow diagram. HIV, human immunodeficiency virus.

A sample flow diagram is shown in Figure 1 and participants' sociodemographic, clinical data, sexual behaviors, and anal douching practice are shown in Table 1. Among 115 participants, the mean age was 28.9 years (SD, 7.4). A majority had either secondary or higher education, and most were unmarried (to a woman). Fifty‐one men (44.3%) were HIV‐infected. gbMSM living with HIV were older than the HIV‐negative group (mean 30.3 vs. 27.7, *p* = 0.011). There were no significant differences in marital status and education level by HIV status. More than two‐thirds (72.5%) were on antiretroviral therapy, and more than half (58.8%) had CD4 count ≤500 cells/μL with a median CD4 percentage of 23.0 (IQR, 18.0–28.0). Slightly more than one‐third were positive for *N. gonorrhea* and other STIs and gbMSM living with HIV were more likely to have STIs than their HIV‐negative counterparts (23.7% vs. 13.0%, *p* = 0.021). More than half (58.8%) reported previous sex work with a median duration of 6 years (IQR, 3–13.0). gbMSM living with HIV had longer duration of sex work as compared to the HIV‐negative group (mean 7.0 vs. 4.0, *p* = 0.048). Compared to the HIV‐negative group, gbMSM living with HIV reported engagement in more frequent casual (66.7% vs. 47.5%, *p* = 0.042), and transactional sex (54.9% vs. 36.1%, *p* = 0.046).

**TABLE 1:** Characteristics stratified by HIV status.

	Total	HIV		
Characteristic	*n* (%) *N* = 115	Positive *N* = 51	Negative *N* = 61	*p*‐value*
Age, mean (SD)	28.9 (7.4)	30.3 (6.7)	27.7 (7.8)	0.011
Age category				
≤ 30	69 (63.9)	28 (54.9)	41 (71.9)	0.066
31+	39 (36.1)	23 (45.1)	16 (28.1)	
Marital status				
Married to a woman	17 (20.0)	10 (23.8)	7 (16.3)	0.386
Single	68 (80.0)	32 (76.2)	36 (83.7)	
Education level				
Primary	10 (11.8)	5 (11.9)	5 (11.6)	0.586
Secondary	34 (40.0)	19 (45.2)	15 (34.9)	
Tertiary	41 (48.2)	18 (42.9)	23 (53.5)	
HIV and related information				
HIV				
Negative	61 (54.5)	–	–	
Positive	51 (45.5)	–	–	
On ART among HIV+	37 (72.5)	37 (72.5)	–	
CD4 count category				
≤500	36 (33.0)	30 (58.8)	–	
500+	73 (67.0)	21 (41.2)	–	
CD4%–Median (IQR)	31.0 (24.0, 40.0)	23.0 (18.0, 28.0)	–	
Undetectable viral load	19 (50.0)	18 (48.6)	–	
STI infection				
No	55 (65.5)	19 (50.0)	36 (78.3)	0.021
Gonorrhea	15 (17.9)	9 (23.7)	6 (13.0)	
Other	14 (16.7)	10 (26.3)	4 (8.7)	
Sexual behaviors and hygiene practices				
Sex work	50 (58.8)	27 (64.3)	23 (53.5)	0.312
Duration of sex work (years), median (IQR)	6.0 (3.0, 13.0)	7.0 (5.5, 14.5)	4.0 (3.0, 12.0)	0.048
Number of sex partners, median (IQR)	6.0 (3.0, 11.0)	9.0 (3.0, 13.0)	5.0 (2.0, 8.0)	0.114
All types of sex	83 (72.2)	41 (80.4)	42 (68.9)	0.165
Regular partners	81 (70.4)	40 (78.4)	41 (67.2)	0.186
Casual partners	63 (54.8)	34 (66.7)	29 (47.5)	0.042
Transactional partners	50 (43.5)	28 (54.9)	22 (36.1)	0.046
Oral sex	85 (73.9)	42 (82.4)	43 (70.5)	0.144
Regular partners	84 (73.0)	41 (80.4)	43 (70.5)	0.228
Casual partners	69 (60.0)	35 (68.6)	34 (55.7)	0.162
Transactional partners	58 (50.4)	30 (58.8)	28 (45.9)	0.173
Insertive sex	85 (73.9)	42 (82.4)	43 (70.5)	0.144
Regular partners	83 (72.2)	40 (78.4)	43 (70.5)	0.339
Casual partners	69 (60.0)	35 (68.6)	34 (55.7)	0.162
Transactional partners	58 (50.4)	31 (60.8)	27 (44.3)	0.081
Receptive sex	85 (73.9)	42 (82.4)	43 (70.5)	0.144
Regular partners	83 (72.2)	40 (78.4)	43 (70.5)	0.339
Casual partners	83 (72.2)	40 (78.4)	43 (70.5)	0.339
Transactional partners	83 (72.2)	40 (78.4)	43 (70.5)	0.339
Sex with condom use	84 (73.0)	41 (80.4)	43 (70.5)	0.228
Regular partners	83 (72.2)	40 (78.4)	43 (70.5)	0.339
Casual partners	67 (58.3)	34 (66.7)	33 (54.1)	0.177
Transactional partners	58 (50.4)	30 (58.8)	28 (45.9)	0.173
Douching practice	54 (63.5)	27 (64.3)	27 (62.8)	0.886

Abbreviations: ART, antiretroviral therapy; CD, cluster of differentiation; HIV, human immunodeficiency virus; IQR, interquartile range; SD, standard deviation; STI, sexually transmitted infection.

* p‐value calculated using Pearson's chi‐squared test or Fisher's exact test for categorical variables and the nonparametric Mann–Whitney U‐test for noncategorical variables.

### 3.2 HPV prevalence and genotype distributions

The overall prevalence of HPV was 51.3% (59/115); the prevalence of HR‐HPV and LR‐HPV were 32.2% and 29.6%, respectively (Table 2). Multiple HPV types were also detected; 28 (24.3%) had multiple HPV infections, 11 (9.6%) had multiple HR‐HPV infections, and 34 (29.6) had LR‐HPV infections. However, there were no cases with HPV‐16 and ‐18 co‐infection. Ten men (8.7%) had HPV‐16 and only 2 (1.7%) had HPV‐18. When stratified by HIV status, the HPV prevalence was 84.3% for gbMSM living with HIV and 24.6% for gbMSM without HIV (*p* < 0.001). Compared to the HIV‐negative group, gbMSM living with HIV were also more likely to have multiple HPV (*p* = 0.011), LR‐HPV (*p* < 0.001), HR‐HPV (*p* < 0.001), multiple HR‐HPV (*p* = 0.001), and HPV‐16 (*p* = 0.041). However, there were no significant differences in the prevalence of the HPV genotype distributions between ≤30 and 31+ years of age (overall HPV prevalence: 58.9% vs. 41.1%, *p* = 0.265).

**TABLE 2:** Anal HPV genotypes stratified by HIV status and age groups.

	HPV		HIV			Age		
Characteristic	*n* (%) *N* = 115		Positive *N* = 51	Negative *N* = 61	*p*‐value*	≤ 30 *N* = 69	31+ *N* = 39	*p*‐value*
Any HPV	59 (51.3)		43 (84.3)	15 (24.6)	<0.001	33 (58.9)	23 (41.1)	0.265
Multiple HPV	28 (24.3)		18 (35.3)	9 (14.8)	0.011	19 (70.4)	8 (29.6)	0.418
LR‐HPV	34 (29.6)		25 (49.0)	8 (13.1)	<0.001	20 (60.6)	13 (39.4)	0.638
HR‐HPV	37 (32.2)		26 (51.0)	10 (16.4)	<0.001	19 (55.9)	15 (44.1)	0.240
Multiple HR‐HPV	11 (9.6)		10 (19.6)	1 (1.6)	0.001	8 (72.7)	3 (27.3)	0.743
HPV‐16	10 (8.7)		8 (15.7)	2 (3.3)	0.041	5 (50.0)	5 (50.0)	0.491
HPV‐18	2 (1.7)		2 (3.9)	0 (0.0)	0.205	1 (50.0)	1 (50.0)	1.000
Any 4‐valent vaccine type^a^	26 (22.6)	26 (44.1)	21 (48.8)	5 (33.3)	0.299	14 (53.8)	12 (46.2)	0.472
Multiple 4‐valent vaccine type^a^	3 (2.6)	3 (5.1)	1 (2.3)	2 (13.3)	0.161	2 (66.7)	1 (33.3)	1.000
Any 9‐valent vaccine type^b^	36 (31.3)	36 (61.0)	27 (62.8)	9 (60.0)	0.848	20 (57.1)	15 (42.9)	0.726
Multiple 9‐valent vaccine type^b^	7 (6.1)	7 (11.9)	5 (11.6)	2 (13.3)	1.000	3 (42.9)	4 (57.1)	0.429

Abbreviations: HPV, human papillomavirus; HIV, human immunodeficiency virus; LR, low risk; HR, high risk;

* *p*‐value calculated using Pearson's chi‐squared test or Fisher's exact test for categorical variables.

^a^ The 4‐valent Gardasil‐41 vaccine (Merck & Co. Inc.) is effective against HPV genotypes 6, 11, 16, and 18.

^b^ The 9‐valent Gardasil‐91 vaccine is effective against HPV genotypes 6, 11, 16, 18, 31, 33, 45, 52, and 58.

**FIGURE 2:**
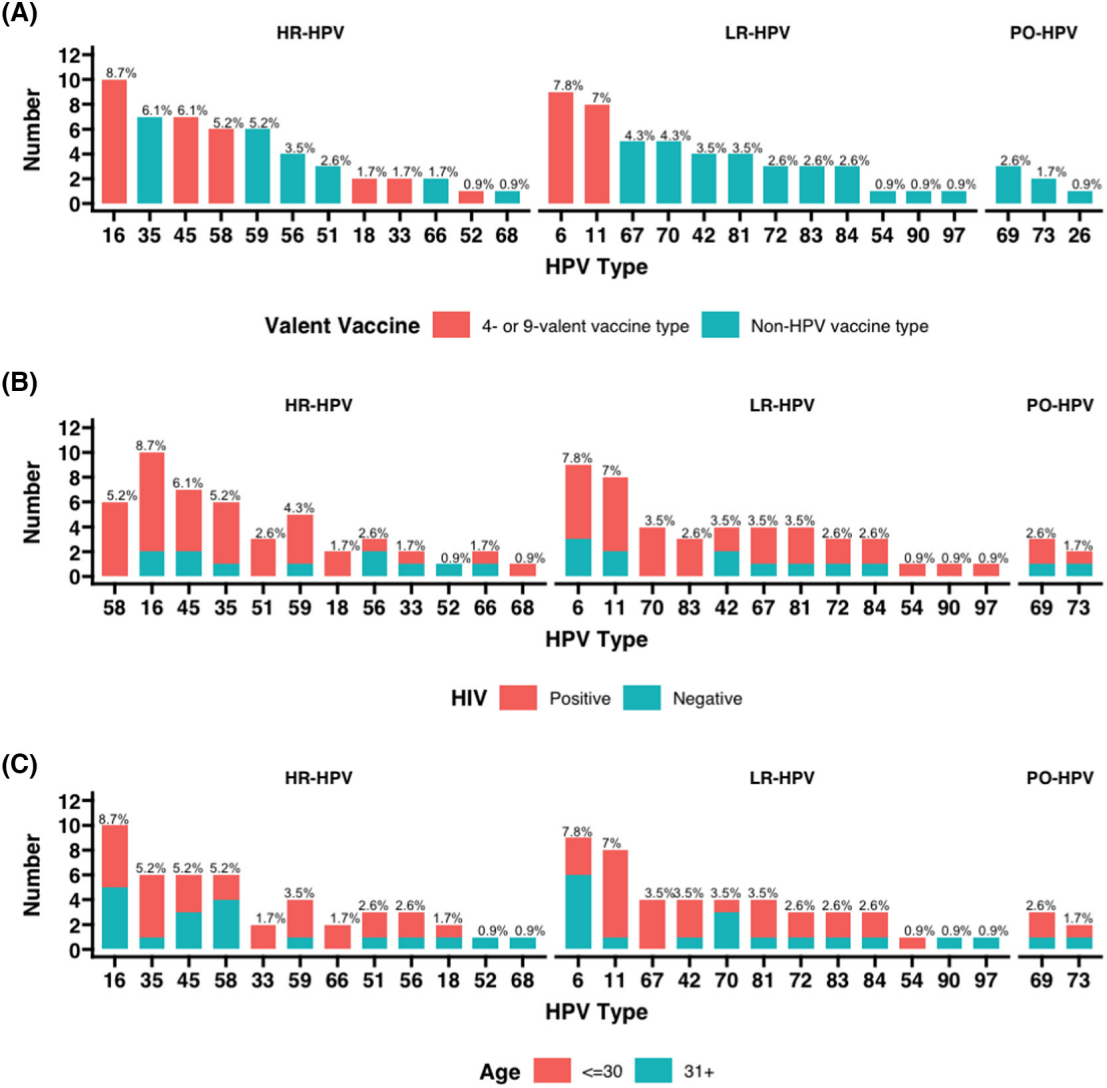
Genotype distributions of anal HPV infections stratified by valent vaccine type, HIV status, and age groups. The prevalence of HPV genotypes is in descending order based on the stratifications. HPV, human papillomavirus; HIV, human immunodeficiency virus; PO, possibly oncogenic.

HPV‐16 was the predominant HR‐HPV type (Figure 2). When stratified by HIV status, HPV‐16, ‐58, and ‐45 were most common among the HIV‐positive group. When stratified by age group, HPV‐16, ‐45, and ‐35 were most frequent among the age group **≤**30 years. Interestingly, all gbMSM infected with HPV‐58, ‐51, ‐18, and ‐68 were HIV‐positive. The vaccine‐preventable HPV genotypes found in this study included HPV‐16, ‐18, ‐33, ‐45, ‐52, and ‐58 for high‐risk types, and HPV‐6, and ‐11 for low‐risk types. If the vaccine was given prior to infection, the 4‐valent and 9‐valent Gardasil vaccines would have prevented type‐specific HPV infections in 26 (44.1%) and 36 (61.0%) gbMSM (Table 2). As for co‐infections with multiple types, 5.1% and 11.9% of gbMSM would have been prevented.

### 3.3 Risk factors for different HPV outcomes

Tables 3 and 4 show findings from multiple logistic regression models assessing the associations between potential risk factors and four HPV outcomes including having any HPV infections, any HR‐HPV infection, and any vaccine‐preventable HPV infections. On unadjusted analyses, anal infections by any HPV and HR‐HPV types were significantly associated with having gonorrhea and being married (cOR: 6.0, 95% CI: 2.0–20.0, *p* = 0.002; cOR: 3.6, 95% CI: 1.1–14.0, *p* = 0.049, respectively). Likewise, anal infection by 9‐valent vaccine‐preventable HPV was significantly associated with being married to a woman. On multivariable analyses, the association between marital status and anal infection by HR‐HPV type (aOR: 8.1, 95% CI: 1.6–52.0, *p* = 0.016) remained significant. HIV was the only independent risk factor that strongly associated with all four HPV outcomes (any HPV – aOR: 23.0, 95% CI: 7.3–86.0, *p* < 0.001; HR‐HPV – aOR: 8.9, 95% CI: 2.8–36.0, *p* < 0.001; 9‐valent type – aOR: 12.0, 95% CI: 3.1–65.0, *p* < 0.001; 4‐valent – aOR: 13.0, 95% CI: 2.8–81.0, *p* = 0.003).

**TABLE 3:** Logistic regression analyses assessing risk factors for HPV outcomes.

	Any HPV					Any HR‐HPV				
	*n* (%)	cOR (95% CI)	*p*‐value	aOR (95% CI)	*p*‐value	*n* (%)	cOR (95% CI)	*p*‐value	aOR (95% CI)	*p*‐value
	*N* = 61					*N* = 38				
Age category										
≤30	33 (58.9)	—		—		19 (55.9)	—		—	
31+	23 (41.1)	1.6 (0.71, 3.5)	0.267	0.5 (0.1, 1.6)	0.238	15 (44.1)	1.6 (0.71, 3.8)	0.242	0.71 (0.2, 2.8)	0.625
Married to a woman	12 (26.1)	2.4 (0.80, 8.2)	0.134	3.5 (0.77, 18)	0.114	11 (40.7)	6.0 (2.0, 20)	0.002	8.1 (1.6, 52)	0.016
Education level										
Primary	6 (13.0)	—				6 (22.2)	—		—	
Secondary	17 (37.0)	0.67 (0.1, 2.8)	0.579			10 (37.0)	0.3 (0.1, 1.2)	0.086	0.2 (0.0, 1.5)	0.142
Tertiary	23 (50.0)	0.85 (0.2, 3.4)	0.823			11 (40.7)	0.2 (0.1, 1.0)	0.055	0.3 (0.0, 1.8)	0.191
HIV status	43 (74.1)	16 (6.6, 45)	<0.001	23 (7.3, 86)	<0.001	26 (72.2)	5.3 (2.3, 13.0)	<0.001	8.9 (2.8, 36.0)	<0.001
STI infection										
No										
Gonorrhea	11 (24.4)	3.6 (1.1, 14)	0.049			6 (21.4)	1.8 (0.52, 5.8)	0.344		
Other	10 (22.2)	3.2 (1.0, 13)	0.072			7 (25.0)	2.7 (0.79, 9.1)	0.110		
Sex worker	45 (76.3)	1.5 (0.67, 3.5)	0.316			26 (70.3)	0.87 (0.4, 2.1)	0.754		
Number of sex partners										
0	17 (28.8)	—				13 (35.1)	—			
1–5	17 (28.8)	1.0 (0.4, 2.5)	>0.999	1.0 (0.1, 9.0)	0.992	11 (29.7)	0.78 (0.3, 2.1)	0.620		
5+	25 (42.4)	1.8 (0.75, 4.6)	0.185	1.3 (0.1, 12)	0.802	13 (35.1)	0.86 (0.3, 2.2)	0.749		
All sex partners	45 (76.3)	1.5 (0.67, 3.5)	0.316			26 (70.3)	0.87 (0.4, 2.1)	0.754		
Insertive sex with partners	46 (78.0)	1.5 (0.67, 3.6)	0.311			27 (73.0)	0.93 (0.4, 2.3)	0.874		
Receptive sex with partners	46 (78.0)	1.5 (0.67, 3.6)	0.311			27 (73.0)	0.93 (0.4, 2.3)	0.874		
Sex with condom use	45 (76.3)	1.4 (0.61, 3.2)	0.424			27 (73.0)	1.0 (0.4, 2.5)	0.991		

Abbreviations: CI, confidence intervals; cOR, crude odds ratio; HIV, human immunodeficiency virus; HPV, human papillomavirus; HR, high risk; STI, sexually transmitted infection.

**TABLE 4:** Logistic regression analyses assessing risk factors for additional HPV outcomes.

	9‐valent vaccine‐preventable HPV					4‐valent vaccine‐preventable HPV				
	*n* (%)	cOR (95% CI)	*p*‐value	aOR (95% CI)	*p*‐value	*n* (%)	cOR (95% CI)	*p*‐value	aOR (95% CI)	*p*‐value
	*N* = 36					*N* = 26				
Age category										
≤30	20 (57.1)	—		—		14 (53.8)	—		—	
31+	15 (42.9)	1.5 (0.66, 3.5)	0.314	1.7 (0.5, 6.3)	0.384	12 (46.2)	1.7 (0.70, 4.3)	0.224	1.9 (0.5, 8.0)	0.375
Married to a woman	10 (33.3)	3.4 (1.2, 11)	0.028			6 (27.3)	1.8 (0.54, 5.5)	0.326		
Education level										
Primary	5 (16.7)	—		—		3 (13.6)	—		—	
Secondary	9 (30.0)	0.4 (0.1, 1.6)	0.169	0.3 (0.0, 2.1)	0.218	6 (27.3)	0.5 (0.1, 2.8)	0.400	0.2 (0.0, 2.1)	0.186
Tertiary	16 (53.3)	0.64 (0.2, 2.6)	0.529	1.4 (0.2, 11)	0.748	13 (59.1)	1.1 (0.3, 5.6)	0.917	2.0 (0.2, 21)	0.538
HIV status	27 (75.0)	6.5 (2.7, 17)	<0.001	12 (3.1, 65)	<0.001	21 (80.8)	7.8 (2.9, 25)	<0.001	13 (2.8, 81)	0.003
STI infection										
No	15 (51.7)	—		—		10 (45.5)	—		—	
Gonorrhea	8 (27.6)	3.0 (0.94, 10)	0.063	1.6 (0.3, 7.8)	0.549	7 (31.8)	3.9 (1.1, 14)	0.028	2.0 (0.4, 10)	0.421
Other	6 (20.7)	2.0 (0.57, 6.8)	0.263	0.78 (0.1, 3.8)	0.765	5 (22.7)	2.5 (0.65, 9.0)	0.164	0.87 (0.1, 4.8)	0.878
Sex worker	19 (63.3)	1.3 (0.54, 3.4)	0.533			16 (72.7)	2.3 (0.82, 7.0)	0.129		
Number of sex partners										
0	8 (22.2)	—		—		5 (19.2)	—		—	
1–5	13 (36.1)	2.0 (0.71, 5.7)	0.201	10 (0.81, 309)	0.107	9 (34.6)	2.1 (0.63, 7.4)	0.241	5.8 (0.4, 188)	0.238
5+	15 (41.7)	2.1 (0.78, 6.0)	0.151	6.9 (0.57, 191)	0.167	12 (46.2)	2.6 (0.87, 9.2)	0.099	7.3 (0.6, 212)	0.163
All sex partners	29 (80.6)	1.9 (0.77, 5.3)	0.180	0.1 (0.0, 10)	0.337	22 (84.6)	2.5 (0.87, 9.2)	0.116		
Insertive sex with partners	30 (83.3)	2.2 (0.84, 6.4)	0.126			22 (84.6)	2.3 (0.78, 8.3)	0.166		
Receptive sex with partners	30 (83.3)	2.2 (0.84, 6.4)	0.126			22 (84.6)	2.3 (0.78, 8.3)	0.166		
Sex with condom use	29 (80.6)	1.8 (0.72, 5.0)	0.224			21 (80.8)	1.7 (0.63, 5.6)	0.317		

Abbreviations: CI, confidence intervals; cOR, crude odds ratio; HIV, human immunodeficiency virus; HPV, human papillomavirus; HR, high risk; STI, sexually transmitted infection.

## 4 DISCUSSION

This study reported the prevalence of anal HPV infections, genotype distributions, and associated risk factors in a Kenyan gbMSM community at high risk of HIV infection and anorectal cancer. We found an overall high prevalence of anal HPV and HIV infections among this group of gbMSM. gbMSM living with HIV were at a higher risk of HPV infections than those without HIV. HPV‐16, ‐35, ‐45, and ‐58 were the predominant HR‐HPV genotypes and multiple HPV infections were also frequent. A significant proportion of gbMSM were infected with vaccine‐preventable HPV genotypes. While HIV was the only significant independent risk factor for all four HPV outcomes assessed in this study, being married to a woman was a strong predictor for having any HR‐HPV infection.

The prevalence of anal HPV infection found in this gbMSM population was lower than the prevalence reported in gbMSM living in South Africa,^15^ Nigeria,^14^ and the Central African Republic,^13^ ranging from 58% to 92%, or in young Black American gbMSM (87%).^22^ Another study in West Africa reported a wide range of HR‐HPV prevalence (33%–71%) suggesting heterogeneity based on the study location.^23^ In contrast, other gbMSM cohorts in China^24^, ^25^ and Thailand^26^, ^27^ reported lower HPV prevalence, ranging between 29% and 56%. Similarly, HIV prevalence reported in this gbMSM population was higher than the prevalence previously reported in Kenya gbMSM (12.3%–43.0%) and in the general Kenyan population (6.1%).^28^ Studies from sub‐Saharan Africa also reported a comparably 5–18 times higher HIV prevalence in gbMSM as compared to the general population.^29^, ^30^ These indicate an overlapping HPV and HIV epidemiology within the gbMSM population in Kenya, despite the varying degree of HPV prevalence in different geographical locations. However, this should not be interpreted as population prevalence as our study was based on a single clinic setting.

Our study showed a much higher prevalence of anal HPV infection in gbMSM living with HIV, which is particularly higher than the reports among gbMSM living with HIV in various settings (72.4% in Central African Republic, 62–82% in China,^31^ 65.3% in Canada,^32^ 65.6% in Brazil,^33^ 74.2% in Taiwan^34^), but lower than the findings from Thai gbMSM cohorts (85%–100%).^27^, ^35^ Anal HPV infections and related diseases have been widely studied among gbMSM population and it has been known that gbMSM living with HIV were at a higher risk of having anal HPV infections than gbMSM without HIV.^10^, ^31^, ^36^, ^37^, ^38^ Our study also confirmed this linkage between HIV status and four HPV outcomes, including vaccine‐preventable HPV infections. Wei et al. also showed in their pooled analysis of 29,900 men that the association between HIV and HPV infections did not limit to high‐risk types but also extended to high‐grade squamous intraepithelial lesions which could eventually lead to anal cancers.^10^ They also reported that HPV prevalence tended to increase with age, but due to the limited sample size, we could not investigate this relationship. Nevertheless, these findings strongly suggest that gbMSM is a potential target group at high risk of HPV infections and should be prioritized when considering the evidence‐based implementation of HPV and anal cancer prevention programs in Kenya.

In our study, HR‐HPV genotype distributions appeared atypical with HPV‐58, ‐45, and 35 being frequent, while HPV‐16 remained the predominant genotype and HPV‐18 was poorly represented in this population. Studies from the Central African Republic and Nigeria showed a similar unusual genotype profile of HPV‐35 and 58 being the most prevalent 9‐valent vaccine HR‐HPV.^13^, ^14^ Interestingly, all gbMSM infected by HPV‐35 in this study as well as in other studies were co‐infected with HIV, suggesting a regional concordance of such unique HPV genotype epidemiology. Although Bouassa and colleagues pointed out that HPV‐16 and ‐18 were less prevalent in young Black American gbMSM, our findings suggested heterogeneity in genotype distributions and Kenya needs evidence in the local context to advocate and promote HPV vaccination within the gbMSM community.

Interestingly, gbMSM married to women were found to be at an increased risk of having HR‐HPV infections. This could be explained in the following way. Within Kenya, gbMSM often have female sex partners which may contrast with gbMSM in other countries. Condom usage among married couples in Kenya remains low due to a number of factors. Examples include the male partners' unwillingness to use condoms could be compounded the female partners' lack of power to negotiate. Additionally, the subsequent higher uptake of contraceptives among married women is an important consideration, especially as access to these medications increases in rural and urban areas. All these factors drive the HIV and HPV epidemiology in this setting.^39^ However, we did not find any studies examining condomless sex and the transmission of HR‐HPV among married gbMSM in Kenya or elsewhere. Despite having relatively higher HPV and HR‐HPV prevalence, HPV and cancer‐related knowledge was low among Kenyan women,^40^ with many reporting no knowledge of HPV as either a sexually transmitted infection or as a cancer‐causing pathogen, and another recent study showed that only a small fraction of these women residing in Nairobi underwent pap smear.^41^ This suggests implications for HPV transmission networks and HR‐HPV genotypes circulating among married couples could also be attributable to the high HR‐HPV prevalence observed among gbMSM who are married to women. Furthermore, lack of education surrounding HPV, HPV‐associated cancers, and the role of HPV vaccination among women within Kenya may also play an integral role in understanding the epidemiology of HR‐HPV infections between married partners, including women with male partners who have sex with men.

Several HPV genotypes included in the prophylactic Gardasil‐9 and ‐4 vaccines were detected in half of HPV‐positive anal samples. The high prevalence of 9‐ and ‐4 valent HPV vaccine types in the anogenital region suggests a potential area of intervention by targeted HPV vaccination to reduce the burden of HPV infections and associated cancer risk among Kenyan gbMSM. The estimated efficacy is based on the prevalence of the strains included in the vaccine and we would expect higher efficacy against the development of precancerous lesions, given that HPV vaccines can protect against certain non‐vaccine HPV types due to cross‐reactivity. Despite an increase in global demand in recent years, the World Health Organization recently implemented a general recommendation that HPV vaccination for boys be suspended as of 2019. This decision may be contextualized on the notion of “limited supply”, where girls and women are prioritized over boys and men.^42^ However, “supply limitations” should not be the reason for not vaccinating the gbMSM population, and increasing manufacturing capacity, even at a modest amount, should be reconsidered.

In addition, a clinical program providing care and support in this population reported more anal cancer and anal diseases detected coupled with high cases of HIV co‐infections in the same cohort.^20^ The program is currently planning HPV screening on selected criteria, which will be embedded in their routine anal health care services for gbMSM communities. Their recent proposal containing an urgent call to provide HPV vaccines to gbMSM communities pointed out an excess in Kenya's stock supply of HPV vaccine due to low uptake among the conventional target groups of AGYW. This highlighted the possibility of procuring required HPV vaccines for the gbMSM community from the available to‐be‐soon‐expired stock.^20^ Hence, this would warrant careful planning and evidence‐based implementation of the gbMSM‐inclusive and targeted HPV vaccination efforts in Kenya.

Our study had some limitations. Indeed, the recruitment of participants from one gbMSM clinic in Nairobi, Kenya, as well as the small sample size of our study population, may have introduced selection and information bias. Hence, the study participants may not represent the Kenyan gbMSM community. On the other hand, the clinic is well‐known across the county and is freely accessible to the gbMSM community. This may not entirely alleviate the bias but could help understand the contextual HPV prevalence in this gbMSM population and inform on the upcoming prevention strategies. In addition, our study has missing values in some key variables including marital status and sex work, which warrants cautious interpretation when asserting corresponding relationship with HPV prevalence.^1^

## 5 CONCLUSION

Prevention of HPV infections and anal cancers through vaccination, as well as early detection and treatment, are on the horizon for Kenya, and promising developments around anal health care including HPV vaccination and anal cancer screening were proposed by The Global Fund to Fight AIDS, Tuberculosis and Malaria. The gbMSM community living in Kenya could be at high risk of having anal HR‐HPV infections, especially among gbMSM living with HIV, and major HR‐HPV genotypes observed are covered by available valent vaccines. Targeted HPV vaccination toward gbMSM living with HIV could well serve as a potential key prevention strategy for this community and should be urgently prioritized.

## AUTHOR CONTRIBUTIONS

**Myo Minn Oo:** Conceptualization (equal); data curation (equal); formal analysis (equal); investigation (equal); methodology (equal); software (equal); validation (equal); visualization (equal); writing – original draft (equal); writing – review and editing (equal). **Samantha MOORE:** Formal analysis (equal); visualization (equal); writing – original draft (equal); writing – review and editing (equal). **Suzanne GIBBONS:** Conceptualization (equal); data curation (equal); funding acquisition (equal); methodology (equal); resources (equal); supervision (equal); writing – review and editing (equal). **Wendy ADHIAMBO:** Data curation (equal); investigation (equal); resources (equal); writing – review and editing (equal). **Peter MUTHOGA:** Data curation (equal); investigation (equal); resources (equal); writing – review and editing (equal). **Naomi SIELE:** Data curation (equal); investigation (equal); resources (equal); writing – review and editing (equal). **Maureen AKOLO:** Data curation (equal); investigation (equal); resources (equal); writing – review and editing (equal). **Henok GEBREBRHAN:** Conceptualization (equal); data curation (equal); investigation (equal); resources (equal); writing – review and editing (equal). **Aida SIVRO:** Conceptualization (equal); data curation (equal); formal analysis (equal); funding acquisition (equal); investigation (equal); methodology (equal); resources (equal); supervision (equal); writing – review and editing (equal). **Blake T. BALL:** Conceptualization (equal); data curation (equal); funding acquisition (equal); investigation (equal); methodology (equal); resources (equal); supervision (equal); writing – review and editing (equal). **Robert R. LORWAY:** Conceptualization (equal); investigation (equal); methodology (equal); resources (equal); writing – review and editing (equal). **Alberto SEVERINI:** Conceptualization (equal); data curation (equal); investigation (equal); methodology (equal); resources (equal); writing – review and editing (equal). **Joshua KIMANI:** Conceptualization (equal); funding acquisition (equal); investigation (equal); methodology (equal); resources (equal); writing – review and editing (equal). **Lyle R. MCKINNON:** Conceptualization (equal); data curation (equal); funding acquisition (equal); investigation (equal); methodology (equal); resources (equal); supervision (equal); validation (equal); writing – original draft (equal); writing – review and editing (equal).

## FUNDING INFORMATION

This work was funded by the Canadian Institutes of Health Research (CIHR), Research Manitoba, and the University of Manitoba. Aida Sivro was supported by a European & Developing Countries Clinical Trials Partnership (EDCTP) Career Development Fellowship (TMA2016 CDF‐1582). L.R.M. was supported by a CIHR New Investigator Award.

## CONFLICT OF INTEREST STATEMENT

There are no conflicts of interest.

## Data Availability

The datasets generated during and/or analyzed during the current study are not publicly available due to the sensitive nature and confidentiality of the data pertaining to the gay, bisexual, and men who have sex with men who were included in the study but anonymized data can be available from the corresponding author on reasonable request.
